# Traditional Chinese Medicine Qingre Huoxue Treatment vs. the Combination of Methotrexate and Hydroxychloroquine for Active Rheumatoid Arthritis: A Multicenter, Double-Blind, Randomized Controlled Trial

**DOI:** 10.3389/fphar.2021.679588

**Published:** 2021-05-25

**Authors:** Xun Gong, Wei-Xiang Liu, Xiao-Po Tang, Jian Wang, Jian Liu, Qing-Chun Huang, Wei Liu, Yong-Fei Fang, Dong-Yi He, Ying Liu, Ming-Li Gao, Qing-Jun Wu, Shi Chen, Zhen-Bin Li, Yue Wang, Yan-Ming Xie, Jun-Li Zhang, Cai-Yun Zhou, Li Ma, Xin-Chang Wang, Chi Zhang, Quan Jiang

**Affiliations:** ^1^ Guang’anmen Hospital China Academy of Chinese Medical Sciences, Beijing, China; ^2^ The First Affiliated Hospital of Anhui University of Chinese Medicine, Hefei, China; ^3^ Guangdong Provincial Hospital of Traditional Chinese Medicine, Guangzhou, China; ^4^ The First Affiliated Hospital of Tianjin University of Traditional Chinese Medicine, Tianjin, China; ^5^ Affiliated Hospital of the Third Military Medical University of the Chinese People’s Liberation Army, Chongqing, China; ^6^ Shanghai Guanghua Hospital of Integrated Traditional Chinese and Western Medicine, Shanghai, China; ^7^ The Affiliated Hospital of Shandong University of Traditional Chinese Medicine, Jinan, China; ^8^ The Affiliated Hospital of Liaoning University of Traditional Chinese Medicine, Shandong, China; ^9^ Peking Union Medical College Hospital, Beijing, China; ^10^ Peking University People’s Hospital, Beijing, China; ^11^ Bethune International Peace Hospital, Shijiazhuang, China; ^12^ Jiangsu Provincial Hospital of Traditional Chinese Medicine, Beijing, China; ^13^ Institute of Basic Research in Clinical Medicine, China Academy of Chinese Medical Sciences, Beijing, China; ^14^ The Fifth Hospital of Xi’an, Xi’an, China; ^15^ Xiyuan Hospital China Academy of Chinese Medical Sciences, Beijing, China; ^16^ China-Japan Friendship Hospital, Beijing, China; ^17^ The Second Affiliated Hospital of Zhejiang University of Chinese Medicine, Hangzhou, China; ^18^ Dongzhimen Hospital Beijing University of Chinese Medicine, Beijing, China

**Keywords:** qingre huoxue decoction, damp-heat-stasis syndrome, active rheumatoid arthritis, comprehensive treatment., randomized controlled trial

## Abstract

Traditional Chinese medicine (TCM) has been used successfully to treat rheumatoid arthritis (RA). Qingre Huoxue treatment (Qingre Huoxue decoction (QRHXD)/Qingre Huoxue external preparation (QRHXEP)) is a therapeutic scheme of TCM for RA. To date, there have been few studies comparing the efficacy and safety of QRHXD and conventional synthetic disease-modifying antirheumatic drugs (csDMARDs) for the treatment of active RA. This was investigated in a multicenter, double-blind, randomized controlled trial involving 468 Chinese patients with active RA [disease activity score (DAS)-28 > 3.2] treated with QRHXD/QRHXEP (TCM group), methotrexate plus hydroxychloroquine [Western medicine (WM) group], or both [integrative medicine (IM) group]. Patients were followed up for 24 weeks. The primary outcome measure was the change in DAS-28 from baseline to 24 weeks. The secondary outcome measures were treatment response rate according to American College of Rheumatology 20, 50, and 70% improvement criteria (ACR-20/50/70) and the rate of treatment-related adverse events (TRAEs). The trial was registered at ClinicalTrials.gov (NCT02551575). DAS-28 decreased in all three groups after treatment (*p* < 0.0001); the score was lowest in the TCM group (*p* < 0.05), while no difference was observed between the WM and IM groups (*p* > 0.05). At week 24, ACR-20 response was 73.04% with TCM, 80.17% with WM, and 73.95% with IM (based on the full analysis set [FAS], *p* > 0.05); ACR-50 responses were 40.87, 47.93, and 51.26%, respectively, (FAS, *p* > 0.05); and ACR-70 responses were 20.87, 22.31, and 25.21%, respectively, (FAS, *p* > 0.05). Thus, treatment efficacy was similar across groups based on ACR criteria. On the other hand, the rate of TRAEs was significantly lower in the TCM group compared to the other groups (*p* < 0.05). Thus, QRHXD/QRHXEP was effective in alleviating the symptoms of active RA—albeit to a lesser degree than csDMARDs—with fewer side effects. Importantly, combination with QRHXD enhanced the efficacy of csDMARDs. These results provide evidence that QRHXD can be used as an adjunct to csDMARDs for the management of RA, especially in patients who experience TRAEs with standard drugs.

Clinical Trial Registration: ClinicalTrials.gov, identifier NCTNCT025515.

## Introduction

Rheumatoid arthritis (RA) is the most common autoimmune inflammatory arthritis in adults. RA has significant negative impacts on the ability of patients to perform daily activities—including work and home tasks—and health-related quality of life, and increases the risk of mortality (Singh et al., 2016). The incidence of RA is about 1% globally (van der Woude and van der Helm-van Mil, 2018) and 0.28–0.40% in China (Chinese Rheumatology Association, 2018), with no fewer than five million people in China living with the disease. In traditional Chinese medicine (TCM), clinical manifestations of active RA such as joint swelling and tenderness, morning stiffness, and local skin redness constitute a “damp-heat-stasis syndrome” that is similar to the active period of RA [i.e., disease activity score (DAS)-28 > 3.2]. TCM has been used successfully to treat RA. Specifically, Qingre Huoxue decoction (QRHXD) may prevent bone destruction by modulating inflammation, and short-term application of a cream prepared from *Tripterygium wilfordii*—the source of a QRHXEP component—was shown to be an effective and safe adjunctive treatment (Jiang et al., 2012; China Association of Chinese Medicine, 2017; Jiao et al., 2019). Oral Chinese medicines were found to relieve joint symptoms with minimal adverse reactions in combination with topical formulations for improved disease control (Xing et al., 2020).

The goals of RA treatment are to achieve disease remission or low disease activity and ultimately control the disease, reduce the disability rate, and improve patients’ quality of life (Smolen et al., 2020). Conventional synthetic disease-modifying antirheumatic drugs (csDMARDs) are the first-line treatment for RA in China and elsewhere (Singh et al., 2016; Chinese Rheumatology Association, 2018; Lau et al., 2019; Smolen et al., 2020). Methotrexate (MTX) is the anchor drug (Pincus et al., 2003) while hydroxychloroquine (HCQ) is mainly used in combination treatment regimens (95%) (Zhang et al., 2013); the utilization rates of these drugs are 55.9 and 30.4%, respectively, (Jin et al., 2017). However, MTX and HCQ have adverse effects such as gastrointestinal discomfort (e.g., nausea and diarrhea) (Giraud et al., 2020), hepatitis, interstitial pneumonitis, cytopenias, and retinopathy (Abbasi et al., 2019); as such, the vast majority of RA patients in China are willing to receive TCM treatment (Lu et al., 2019).

Qingre Huoxue treatment (QRHXD plus QRHXEP) is a therapeutic scheme of TCM for RA that have been routinely used in clinical practice for at least 40 years at Guang’anmen Hospital, China Academy of Chinese Medical Sciences. However, there have been few blinded trials systematically comparing the efficacy and safety of Qingre Huoxue treatment and csDMARDs in RA treatment. Thus, the aim of this investigation was to evaluate the effects of Qingre Huoxue treatment vs. MTX plus HCQ in patients with active RA.

## Materials and Methods

### Trial Design

This multicenter, double-blind, randomized controlled clinical trial has been registered at ClinicalTrials.gov (NCTNCT02551575). The study was conducted in accordance with the principles outlined in the Declaration of Helsinki, Good Clinical Practice Guidelines of the International Conference on Harmonization, and local regulatory requirements. This study followed CONSORT. The protocol was approved by the ethics committee at Guang’anmen Hospital, China Academy of Chinese Medical Sciences (no. 2013EC122). A study period of 24 weeks was selected based on pilot clinical trials.

### Participants

Men and women aged 18–65 years were considered for enrollment if they met the American College of Rheumatology (ACR) criteria for RA (Arnett et al., 1988; Lu and Jiao, 1996; Aletaha et al., 2010) as well as TCM criteria for the diagnosis of RA “damp-heat-stasis syndrome” (Zheng, 2002) (Supplementary Material). The trial was conducted at 16 hospitals. An independent committee monitored the trial for safety and scientific integrity. The inclusion criteria were as follows: 1) DAS-28 score > 3.2; 2) patients were taking csDMARDs for at least 3 months at a stable dose, and continued the same treatment for the duration of the present study; and 3) patients voluntarily participated in the study and signed an informed consent form. Exclusion criteria were as follows: 1) patients with skin burst or allergies; 2) patients with cancer or other malignant diseases such as cardiovascular, hematopoietic, liver, or kidney disease or psychopathy; 3) patients with active or chronic infection, including HIV, hepatitis C virus, hepatitis B virus, or tuberculosis; 4) treatment with *Tripterygii*, glucocorticoid, or biologic DMARDs in the prior 3 months; 5) previous treatment with MTX or HCQ; and 6) patients with retinopathy. Patients who met the following criteria were not allowed to continue receiving the therapy as part of the study, but were followed up until the end of the trial: 1) patients who experienced intolerable adverse events (AEs), complications, or physiologic changes, and had discontinued the study drug for more than 14 days; 2) patients who demonstrated poor compliance (the number and course of medication were not between 80 and 120%); 3) patients who were unwilling to continue participating in the study or were lost to follow-up; and 4) patients who used a nonprescribed range of combined medications that could affect the efficacy and safety assessments.

### Treatment

Eligible patients obtained sequence numbers from the trial coordinator and were allocated to 1 of the 3 following groups by computer-generated sequence randomization: 1) TCM group (TCM plus MTX and HCQ placebos); 2) Western medicine (WM) group (12.5 mg MTX once weekly plus 200 mg HCQ twice daily combined with TCM placebo); and 3) integrative medicine (IM) group (TCM combined with 12.5 mg MTX once weekly plus 200 mg HCQ twice daily). MTX and HCQ tablets were obtained from Shanghai Xinyi Pharmaceutical Co. (Shanghai, China) and Shanghai Zhongxi Pharmaceutical Co. (Shanghai, China), respectively, and were taken orally. TCM Qingre Huoxue treatment for RA “damp-heat-stasis syndrome” with QRHXD and QRHXEP optimized by the Guang’anmen Hospital China Academy of Chinese Medical Sciences were used in this study. QRHXD was processed into granules that were packaged in a tin foil bag by Sichuan New Green Pharmaceutical Technology Development Co. (Chengdu, China). QRHXEP was processed into a gel formulation and packaged in a plastic tube at Guang’anmen Hospital China Academy of Chinese Medical Sciences (batch no. 15011303). QRHXD was taken twice daily—at breakfast and 0.5 h after dinner—for 24 weeks (1 bag boiled in water for each dose). QRHXEP was applied topically once daily 1 h after dinner for 12 weeks [20 g (1 stick) daily in weeks 1–4 and 10 g (half a stick) daily in weeks 5–12] to the skin surface of affected joints, while avoiding the face and temporomandibular joint.

QRHXD has 12 components including animal drug wugong (*Centipede* [4 g]) and the botanical drugs species or TCM plant preparations tufuling (*Smilax glabra Roxb* [30 g]), yinhua (Lonicera japonica Thunb [30 g]), huangqi (*Astragalus mongholicus* [30 g]), chaocangzhu (bran-fried *Atractylodes chinensis* [15 g]), huangbo (*Phellodendron amurense* [9 g]), chishao (*Paeonia lactiflora* [15 g]), bixie (*Dioscoreae hypoglaucae rhizoma* [15 g]), danshen (*Salvia miltiorrhiza* [15 g]), ezhu (*Curcuma zedoaria* [9 g]), qingfengteng (*Sinomenium acutum* (Thunb.) [15 g]), and fengfang (*Nidus vespae* [5 g]). The granules were packaged as 10-g bags, with each bag containing 96 g of crude product. QRHXEP is composed of six botanical drugs including leigongteng (*T. wilfordii* [120 g]), chuanxiong (*Ligusticum wallichii* [60 g]), baizhi (*Angelica dahurica* [60 g]), dahuang (*Rheum officinale* [30 g]), ruxiang (*Boswellia carterii* [30 g]), and bohe (*Fructus forsythiae* [30 g]), as well as the resin moyao (*Myrrh* [30 g]) and mineral mangxiao (*Mirabilite* [120 g]). *T. wilfordii*, *R. officinale*, and *A. dahurica* were refluxed in a volume of 75% ethanol eight times the combined weight of the three components for 1 h. The extracts were combined and filtered, and the ethanol was separated from the filtrate and set aside with the residue stored in another container. *L. wallichii*, *B. carterii*, and myrrh were added to a volume of water 6 times the combined weight of the three components and the volatile oils were extracted for 8 h, and the liquid was filtered and set aside. The residues of *L. wallichii*, *B. carterii*, and *Myrrh* were combined with those of *T. wilfordii*, *R. officinale*, and *A. dahurica*; *Mirabilite* was added along with a 6 × volume of water. The mixture was decocted for 1 h and filtered; the filtrate was combined with the extract liquids that had been set aside, concentrated to a relative density of 1.055–1.075 (75°C), and filtered through a 100-mesh sieve and set aside. *F. forsythiae* was steam-distilled and frozen, and partially decapitated to obtain 0.3 ml peppermint oil. The volatile oils extracted from *L. wallichii*, *B. carterii*, and *Myrrh* were combined with the peppermint oil and ethyl benzoate and dissolved in ethanol. Carboxymethyl cellulose sodium and glycerol were added with stirring. Water was then added to the concentrated extract liquid up to a weight of 1,000 g with continuous stirring until a gel was obtained. The names of the TCM ingredients in Chinese and English are listed in Supplementary Material.

QRHXD placebo granules were composed of lactose, starch, edible colorant, and bitter taste agents packaged in a tin foil bag identical to the one containing QRHXD granules and had the same color, texture, taste, and smell as the actual medicine. The QRHXEP placebo gel was prepared from viscous agent with cane sugar color added so that it had the same color, texture, and smell as the actual QRHXEP gel. The MTX and HCQ placebo tablets were composed of lactose, starch, carboxymethyl starch sodium, and magnesium stearate and had the same color, texture, taste, and smell as the actual drugs.

The quality of the TCM granules and gel was evaluated according to the 2005 Chinese pharmacopoeia (Chinese Pharmacopoeia Commission, 2005). Before the start of the trial, the TCM granules and gel were tested for heavy metals, microbial contamination, and residual pesticides, and were determined to meet the safety standards in China. Laboratory personnel were blinded to the identity of the TCM granules and gel. QRHXD and QRHXEP and their placebos were prepared by the same manufacturer. The medicines were distributed to the 16 study sites from the same source.

### Measurements

The patients were observed for 24 weeks. The primary outcome measure was the change in DAS-28 from baseline to week 24, and the secondary outcome measures were ACR-20, ACR-50, and ACR-70 (ACR criteria for 20, 50, and 70% improvement, respectively), which were evaluated at week 24. DAS-28 was based on erythrocyte sedimentation rate (ESR), 28 tender and swollen joint counts (TJC and SJC, respectively), general health (GH; patient assessment of disease activity according to a 100-mm visual analogue scale with 0 = best and 100 = worst), and levels of acute phase reactant (ESR [mm/h]), and was calculated as 0.56 × √(TJC28) + 0.28 × √(SJC28) + 0.014×GH + 0.70 × ln(ESR) (Wells et al., 2009). The ACR-20/50/70 response was defined as ≥ 20%/50%/70% improvement in both the TJC and SJC and ≥ 20%/50%/70% improvement in three of the five other core measures (resting pain, patient’s global assessment, physician’s global assessment, Health Assessment Questionnaire [HAQ] score, and ESR/C-reactive protein ratio) (Felson et al., 1995). Physical function was assessed at baseline and at week 24 with the HAQ (Bruce and Fries, 2005). Safety was assessed based on routine blood and urine tests, liver (alanine aminotransferase and aspartate aminotransferase) and renal (blood urea nitrogen and creatinine) function, electrocardiogram, and treatment-related (TR)AEs at week 24.

### Sample Size

The sample size was determined by the primary outcome. Assume that the ratio of the three groups is 1:1:1, using a two-sided test with a significance level (α) of 0.05 and a power (1-β) of 0.90, and the required sample size is estimated by SAS 9.4 software for 129 cases in each group. Allowing for a dropout of about 20%, and the TCM group, WM group and the IM group each required 156 cases, a total of 468 cases.

### Statistical Analysis

Intention-to-treat (ITT) approach was performed using in all of analyses. Continuous data was presented as mean (SD), and categorical data was presented as numbers and/or percentages. A one-way ANOVA or Kruskal–Wallis rank test combined with the t or Wilcoxon rank test for post hoc testing was used in analyzing the continuous data. The chi-square or Fisher exact test was used in analyzing the categorical data. The Kruskal–Wallis test combined with the Wilcoxon rank test for post hoc testing was used in analyzing the ordinal data. A one-way ANOVA or Kruskal–Wallis rank test combined with the t or Wilcoxon rank test for post hoc testing was used in analyzing difference value (change before and after the treatment) among three groups. For this three-arm clinical study (groups TCM, WM, and IM), we care about whether there are differences in the efficacy of the three groups. Therefore, after completing the difference test for the three groups, it is necessary to further conduct multiple comparisons between the three groups. In order to avoid false positive results, it is necessary to adjust the test level in the multiple comparison. The Bonferroni method is one of the classic methods for the test level adjustment. The processing method is as follows: the accepted test level is divided by the times of multiple comparisons, and then used as the adjusted test level for multiple comparisons. In this paper, it is necessary to complete the pairwise comparison between the three groups, TCM-WM, TCM-IM, and WM-IM, the test level is adjusted to 0.0167 (0.05/3). That is, the analysis method for two independent samples is used to test the differences between the TCM-WM, TCM-IM, and WM-IM groups. At this time, only when *p* < 0.0167, the difference is considered to be statistically significant. This method can effectively control the generation of false positive results. Statistical analyses were performed using SAS v9.4 software (SAS Institute, Cary, NC, United States).

## Results

### Phytochemical Analysis

Preparation and assay methods of QRHXD and QRHXEP are based on the Pharmaceutical standards of the Ministry of Health of the People’s Republic of China (Pharmacopoeia of the people’s Republic of China [2010]). The chemical profiling of QRHXD and QRHXEP was detected using LC/MS/MS method, and the details were provided in Supplementary Material.

### Study Participants

**FIGURE 1:**
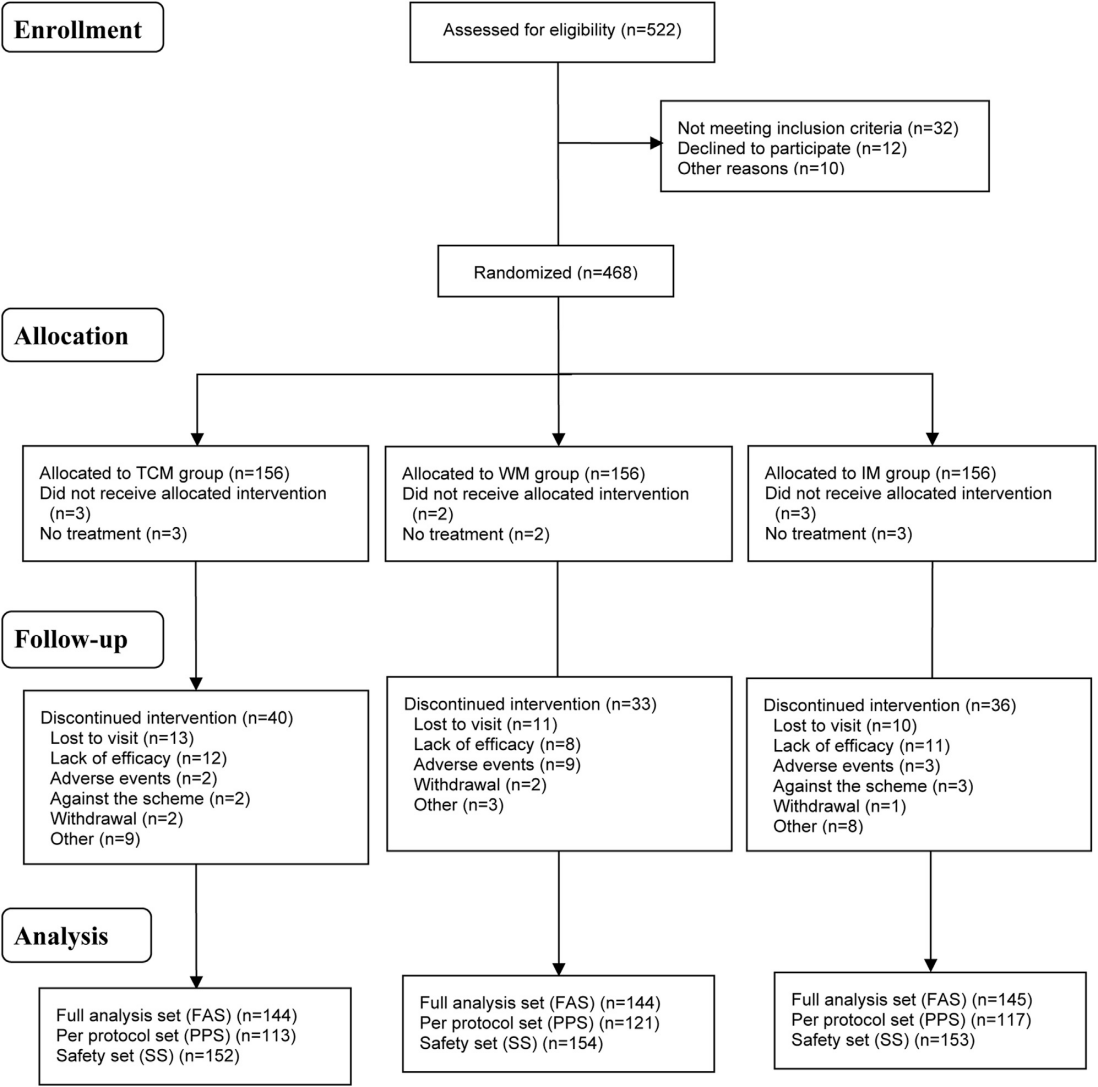
Flowchart of patient selection and treatment group allocation.

Subjects were recruited at 16 medical research centers nationwide (Supplementary Material) from November 2013 to November 2015; the 24-week clinical observation period for all patients was completed in May 2016. A total of 522 patients were screened and 468 were enrolled (156 per group); treatment was discontinued for 109 patients (TCM group, *n =* 40; WM group, *n =* 33; IM group, *n =* 36) and 8 cases were excluded (TCM group, *n =* 3; WM group, *n =* 2; IM group *n =* 3) (Figure 1). There were no significant differences in baseline characteristics between the three groups (Table 1).

**TABLE 1:** Baseline characteristics of the study population (analysis based on full analysis set or safety set).

Characteristic	TCM group		WM group		IM group		P Value
	N (missing)	Mean (SD)/n (%)	N (missing)	Mean (SD)/n (%)	N (missing)	Mean (SD)/n (%)	
Age, years	139 (5)	47.86 (10.70)	139 (5)	48.74 (10.76)	141 (4)	48.11 (10.64)	0.7506
Male sex, n (%)	144 (0)	13 (9.03)	144 (0)	22 (15.28)	145 (0)	25 (17.24)	0.1082
Height, cm	144 (0)	160.43 (5.62)	143 (1)	161.05 (6.04)	145 (0)	161.96 (7.07)	0.2170
Weight, kg	144 (0)	59.39 (10.75)	143 (1)	60.21 (10.35)	145 (0)	60.16 (11.60)	0.7339
SBP, mmHg	149 (3)	121.38 (14.08)	153 (1)	120.99 (14.33)	150 (3)	121.39 (12.74)	0.9124
DBP, mmHg	149 (3)	77.06 (8.79)	153 (1)	77.37 (8.68)	150 (3)	77.19 (9.16)	0.8919
HR, bpm	148 (4)	78.91 (11.68)	150 (4)	76.92 (10.81)	145 (8)	78.10 (9.19)	0.1974
Respiration, times/min	146 (6)	18.42 (1.37)	150 (4)	18.65 (1.37)	147 (6)	18.58 (1.47)	0.3916
Duration, months	141 (3)	21.60 (17.40)	141 (3)	22.17 (19.21)	140 (5)	21.59 (17.78)	0.9832
Patients receiving other drugs, n (%)	140 (4)	66 (51.97)	133 (11)	62 (49.21)	139 (6)	56 (44.44)	0.4804
RF	127 (17)	280.81 (465.01)	127 (17)	321.01 (566.27)	129 (16)	222.66 (436.05)	0.7110
Anti-CCP	76 (68)	523.94 (797.23)	60 (84)	458.42 (756.70)	68 (77)	465.16 (651.49)	0.6533
ESR	140 (4)	42.54 (26.80)	144 (0)	41.68 (27.35)	143 (2)	42.25 (26.37)	0.9437
CRP	143 (1)	20.16 (27.38)	137 (7)	19.17 (26.40)	139 (6)	16.94 (19.76)	0.7649
Resting pain (VAS score, mm)	143 (1)	58.32 (17.11)	144 (0)	54.84 (19.01)	144 (1)	54.70 (18.76)	0.1660
Patient’s global assessment (VAS score, mm)	143 (1)	59.60 (17.62)	144 (0)	56.91 (19.01)	144 (1)	60.22 (19.54)	0.2638
Physician’s global assessment (VAS score, mm)	143 (1)	58.95 (15.18)	144 (0)	54.92 (17.41)	144 (1)	56.41 (18.61)	0.0899
Tender joint count	143 (1)	10.59 (6.62)	144 (0)	9.86 (5.88)	144 (1)	10.56 (6.95)	0.8209
Swollen joint count	143 (1)	7.41 (5.25)	144 (0)	6.80 (5.09)	144 (1)	7.40 (5.31)	0.4422
HAQ score	143 (1)	1.12 (0.65)	144 (0)	0.98 (0.64)	144 (1)	1.03 (0.65)	0.1328

Data are presented as mean (SD) unless otherwise indicated.

CCP, cyclic citrullinated peptide; CRP, C-reactive protein; DBP, diastolic blood pressure; ESR, erythrocyte sedimentation rate; HAQ, Health Assessment Questionnaire; HR, heart rate; IM, integrative medicine; RF, rheumatoid factor; SBP, systolic blood pressure; SD, standard deviation; TCM, traditional Chinese medicine; VAS, visual analog scale; WM, Western medicine.

### Primary Outcome Measure (Disease Activity Score-28)

**FIGURE 2:**
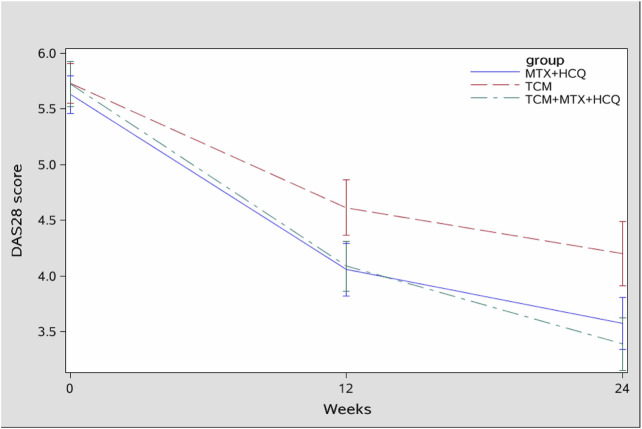
Change in DAS-28 score (full analysis set). TCM, TCM group; MTX+HCQ, WM group; TCM+MTX+HCQ, IM group.

There was no significant difference in DAS-28—the primary outcome measure—among the three groups at baseline (Tables 2, 3). At week 24, DAS-28 was decreased in all three groups compared to the baseline score (*p* < 0.0001). The rank order of DAS-28 at week 24 was IM < WM < TCM (*p* < 0.05) (Tables 4, 5); the score was decreased to 4.20 ± 1.56 in the TCM group, 3.58 ± 1.28 in the WM group, and 3.39 ± 1.27 in the IM group (all p < 0.0001 vs. baseline). The difference value of DAS-28 (i.e., change in score from baseline to week 24) was greater in the IM (2.10 ± 1.12) and WM (2.24 ± 1.40) groups than in the TCM group (1.60 ± 1.17) (p < 0.05), while no difference was observed between the former two groups (*p* > 0.05) (Figure 2).

**TABLE 2:** Distribution of DAS-28 scores and their decline in patients (analysis based on full analysis set).

DAS 28	TCM group			WM group			IM group			*P* Value
	N (missing)	Mean (SD)	*P* Value	N (missing)	Mean (SD)	*P* Value	N (missing)	Mean (SD)	*P* Value	
DAS-28 score										
0 weeks	143 (1)	5.73 (1.08)	−	144 (0)	5.63 (1.02)	−	143 (2)	5.72 (1.23)	−	0.7013
12 weeks	127 (17)	4.61 (1.42)	<0.0001	124 (20)	4.06 (1.32)	<0.0001	129 (16)	4.09 (1.27)	<0.0001	0.0011*
24 weeks	115 (29)	4.20 (1.56)	<0.0001	120 (24)	3.58 (1.28)	<0.0001	113 (32)	3.39 (1.27)	<0.0001	0.0003*
DAS-28 difference value										
12 weeks	127 (17)	1.07 (1.11)	−	124 (20)	1.61 (1.12)	−	128 (17)	1.63 (1.29)	−	0.0005*
24 weeks	115 (29)	1.60 (1.17)	−	120 (24)	2.10 (1.12)	−	112 (33)	2.24 (1.40)	−	0.0006*

*p < 0.05.

DAS, disease activity score; IM, integrative medicine; SD, standard deviation; TCM, traditional Chinese medicine; WM, Western medicine.

**TABLE 3:** Distribution of DAS-28 scores and their decline in patients (analysis based on per protocol set).

DAS 28	TCM group			WM group			IM group			*P* Value
	N (missing)	Mean (SD)	*P* Value	N (missing)	Mean (SD)	*P* Value	N (missing)	Mean (SD)	*P* Value	
DAS-28 score										
0 weeks	113 (0)	5.82 (1.05)	−	121 (0)	5.68 (0.99)	−	116 (1)	5.65 (1.11)	−	0.4055
12 weeks	108 (5)	4.64 (1.45)	<0.0001	118 (3)	4.05 (1.32)	<0.0001	115 (2)	4.10 (1.26)	<0.0001	0.0016*
24 weeks	113 (0)	4.21 (1.57)	<0.0001	119 (2)	3.57 (1.28)	<0.0001	111 (6)	3.41 (1.27)	<0.0001	0.0005*
DAS-28 difference value										
12 weeks	108 (5)	1.17 (1.03)	−	118 (3)	1.64 (1.11)	−	114 (3)	1.57 (1.14)	−	0.0031*
24 weeks	113 (0)	1.62 (1.18)	−	119 (2)	2.10 (1.13)	−	110 (7)	2.23 (1.37)	−	0.0011*

*p < 0.05.

DAS, disease activity score; IM, integrative medicine; SD, standard deviation; TCM, traditional Chinese medicine; WM, Western medicine.

**TABLE 4:** Comparisons of the decline in DAS-28 score between treatment groups (analysis based on full analysis set).

DAS 28	TCM vs. MTX + HCQ		MTX + HCQ vs. TCM + MTX + HCQ		TCM vs. TCM + MTX + HCQ	
	Statistic (Z/t)	*P* Value	Statistic (Z/t)	*P* Value	Statistic (Z/t)	*P* Value
DAS-28 score						
0 weeks	0.79 (t)	0.4283	0.83 (Z)	0.4037	0.04 (t)	0.9656
12 weeks	3.21 (t)	0.0015*	−0.18 (t)	0.8576	3.12 (t)	0.0020*
24 weeks	2.93 (Z)	0.0034*	1.12 (t)	0.2640	−3.85 (Z)	0.0001*
DAS-28 difference value						
12 weeks	3.50 (Z)	0.0005*	0.27 (Z)	0.7881	−3.20 (Z)	0.0014*
24 weeks	−3.28 (Z)	0.0010*	0.53 (Z)	0.5978	−3.74 (t)	0.0002*

*p < 0.05.

DAS, disease activity score; HCQ, hydroxychloroquine; MTX, methotrexate; TCM, traditional Chinese medicine.

**TABLE 5:** Comparisons of the decline in DAS-28 score between treatment groups (analysis based on per protocol set).

DAS 28	TCM vs. MTX + HCQ		MTX + HCQ vs. TCM + MTX + HCQ		TCM vs. TCM + MTX + HCQ	
	Statistic (Z/t)	*P* Value	Statistic (Z/t)	*P* Value	Statistic (Z/t)	*P* Value
DAS-28 score						
0 weeks	1.11 (t)	0.2668	0.18 (t)	0.8608	1.21 (t)	0.2294
12 weeks	3.20 (t)	0.0016*	−0.30 (t)	0.7623	2.98 (t)	0.0032*
24 weeks	2.99 (Z)	0.0028*	0.91 (t)	0.3622	−3.68 (Z)	0.0002*
DAS-28 difference value						
12 weeks	−3.17 (Z)	0.0015*	−0.49 (Z)	0.6240	−2.67 (Z)	0.0075*
24 weeks	−3.13 (Z)	0.0017*	0.50 (Z)	0.6198	−3.57 (t)	0.0004*

*p < 0.05.

DAS, disease activity score; HCQ, hydroxychloroquine; MTX, methotrexate; TCM, traditional Chinese medicine.

### Secondary Outcome Measures

#### American College of Rheumatology-20/50/70

There was no significant difference in ACR-20, ACR-50, and ACR-70 between the three groups at 24 weeks (*p* > 0.05) based on the full analysis set (FAS) (Tables 6, 7). ACR-20 and ACR-70 responses at 12 weeks were lower in the TCM group than in the WM and IM groups (*p* < 0.05). There was no significant difference in ACR-50 between the WM and IM groups at 12 weeks (*p* > 0.05). At 24 weeks, there were no differences in ACR responses among the TCM, WM, and IM groups (ACR-20: 73.04, 80.17, and 73.95%, respectively, *p* = 0.3764; ACR-50: 40.87, 47.93, and 51.26%, respectively, *p* = 0.2674; ACR-70: 20.87, 22.31, and 25.21%, respectively, *p* = 0.7218).

**TABLE 6:** ACR-20, ACR-50, and ACR-70 measurements (analysis based on full analysis set).

ACR	TCM group		WM group		IM group		*P* Value
	N (missing)	Effective no. (%)	N (missing)	Effective no. (%)	N (missing)	Effective no. (%)	
ACR-20							
12 weeks	135 (9)	59 (43.70)	129 (15)	77 (59.69)	135 (10)	81 (60.00)	0.0092*
24 weeks	115 (29)	84 (73.04)	121 (23)	97 (80.17)	119 (26)	88 (73.95)	0.3764
ACR-50							
12 weeks	135 (9)	25 (18.52)	129 (15)	33 (25.58)	135 (10)	33 (24.44)	0.3362
24 weeks	115 (29)	47 (40.87)	121 (23)	58 (47.93)	119 (26)	61 (51.26)	0.2674
ACR-70							
12 weeks	135 (9)	5 (3.70)	129 (15)	13 (10.08)	135 (10)	15 (11.11)	0.0578
24 weeks	115 (29)	24 (20.87)	121 (23)	27 (22.31)	119 (26)	30 (25.21)	0.7218

*p < 0.05.

ACR-20/-50/-70, American College of Rheumatology 20%/50%/70% improvement criteria; IM, integrative medicine; TCM, traditional Chinese medicine; WM, Western medicine.

**TABLE 7:** ACR-20, ACR-50, and ACR-70 measurements (analysis based on per protocol set).

ACR	TCM group		WM group		IM group		*P* Value
	N (missing)	Effective no. (%)	N (missing)	Effective no. (%)	N (missing)	Effective no. (%)	
ACR-20							
12 weeks	113 (0)	52 (46.02)	121 (0)	74 (61.16)	117 (0)	73 (62.39)	0.0205*
24 weeks	113 (0)	83 (73.45)	120 (1)	96 (80.00)	117 (0)	87 (74.36)	0.4431
ACR-50							
12 weeks	113 (0)	21 (18.58)	121 (0)	31 (25.62)	117 (0)	29 (24.79)	0.3832
24 weeks	113 (0)	46 (40.71)	120 (1)	58 (48.33)	117 (0)	60 (51.28)	0.2540
ACR-70							
12 weeks	113 (0)	5 (4.42)	121 (0)	13 (10.74)	117 (0)	12 (10.26)	0.1619
24 weeks	113 (0)	23 (20.35)	120 (1)	27 (22.50)	117 (0)	29 (24.79)	0.7237

*p < 0.05.

ACR-20/-50/-70, American College of Rheumatology 20%/50%/70% improvement criteria; IM, integrative medicine; TCM, traditional Chinese medicine; WM, Western medicine.

Based on the per protocol set (PPS), ACR-20 response at 12 weeks was significantly lower in the TCM group than in the WM and IM groups (*p* < 0.05) (Tables 8, 9). There was no significant difference in ACR-50 and ACR-70 between the latter two groups at 12 weeks (*p* > 0.05). Similarly, at 24 weeks there were no differences in ACR responses among the TCM, WM, and IM groups (ACR-20: 73.45, 80.00, and 74.36%, respectively, *p* = 0.4431; ACR-50: 40.71, 48.33, and 51.28%, respectively, *p* = 0.2540; ACR-70: 20.35, 22.50, and 24.79%, respectively, *p* = 0.7237).

**TABLE 8:** Comparisons of ACR-20, ACR-50, and ACR-70 standard remission between treatment groups (analysis based on full analysis set).

ACR	TCM vs. MTX + HCQ		MTX + HCQ vs. TCM + MTX + HCQ		TCM vs. TCM + MTX + HCQ	
	Statistic (χ^2^)	*P* Value	Statistic (χ^2^)	*P* Value	Statistic (χ^2^)	*P* Value
ACR-20						
12 weeks	6.75(χ2)	0.0094*	0.00(χ2)	0.9590	7.18(χ2)	0.0074*
24 weeks	1.67(χ2)	0.1958	1.31(χ2)	0.2520	0.02(χ2)	0.8752
ACR-50						
12 weeks	1.92(χ2)	0.1659	0.05(χ2)	0.8311	1.41(χ2)	0.2358
24 weeks	1.19(χ2)	0.2750	0.27(χ2)	0.6063	2.54(χ2)	0.1109
ACR-70						
12 weeks	4.22(χ2)	0.0400*	0.07(χ2)	0.7851	5.40(χ2)	0.0201*
24 weeks	0.07(χ2)	0.7876	0.28(χ2)	0.5981	0.62(χ2)	0.4308

*p < 0.05.

ACR-20/-50/-70, American College of Rheumatology 20%/50%/70% improvement criteria; HCQ, hydroxychloroquine; MTX, methotrexate; TCM, traditional Chinese medicine.

**TABLE 9:** Comparisons of ACR-20, ACR-50, and ACR-70 standard remission between treatment groups (analysis based on per protocol set).

ACR	TCM vs. MTX + HCQ		MTX + HCQ vs. TCM + MTX + HCQ		TCM vs. TCM + MTX + HCQ	
	Statistic (χ^2^)	P Value	Statistic (χ^2^)	P Value	Statistic (χ^2^)	P Value
ACR-20						
12 weeks	5.39(χ2)	0.0203*	0.04(χ2)	0.8445	6.21(χ2)	0.0127*
24 weeks	1.40(χ2)	0.2364	1.07(χ2)	0.3006	0.02(χ2)	0.8755
ACR-50						
12 weeks	1.67(χ2)	0.1958	0.02(χ2)	0.8823	1.30(χ2)	0.2543
24 weeks	1.37(χ2)	0.2419	0.21(χ2)	0.6499	2.59(χ2)	0.1078
ACR-70						
12 weeks	3.29(χ2)	0.0699	0.02(χ2)	0.9024	2.86(χ2)	0.0910
24 weeks	0.16(χ2)	0.6901	0.17(χ2)	0.6787	0.65(χ2)	0.4218

*p < 0.05.

ACR-20/-50/-70, American College of Rheumatology 20%/50%/70% improvement criteria; HCQ, hydroxychloroquine; MTX, methotrexate; TCM, traditional Chinese medicine.

#### Safety

No patients died during the study period. More patients in the WM group (*n* = 12) discontinued treatment because of TRAEs than in the IM (*n* = 3) and TCM (*n* = 2) groups (*p* < 0.05) (Tables 10, 11). Fewer patients in the TCM group reported gastrointestinal discomfort (*n* = 4) compared to the WM group (n = 18) and IM group (*n* = 16) (*p* < 0.05). Nausea occurred in one patient treated with TCM compared to eight patients in the WM group and 10 in IM the group (*p* < 0.05). Bacteriuria was also reported at a lower rate in patients treated with TCM (*n* = 8 vs. 20 with WM and 19 with IM) (*p* < 0.05). There was no significant difference in the frequency of other TRAEs among the groups. The overall rate of TRAEs was lower in the TCM group than in the other two groups (*p* < 0.05).

**TABLE 10:** Adverse events (analysis based on safety set).

Adverse event	TCM group (*n* = 152)	WM group (*n* = 154)	IM group (*n* = 153)	*P* Value
Deaths	0	0	0	
SAEs	0	0	0	
Discontinuation due to AE	2 (1.32)	12 (7.79)	3 (1.96)	0.0042*
Discontinuation due to SAEs	0	0	0	
Gastrointestinal	4 (2.63)	18 (11.69)	16 (10.46)	0.0078*
Nausea	1 (0.66)	8 (5.19)	10 (6.54)	0.0261*
Vomit	0 (0.00)	5 (3.25)	3 (1.96)	0.1063
Hiccup	0 (0.00)	1 (0.65)	0 (0.00)	1.0000
Abdominal pain	0 (0.00)	1 (0.65)	0 (0.00)	1.0000
Stomachache	1 (0.66)	2 (1.30)	0 (0.00)	0.6638
Diarrhea	0 (0.00)	1 (0.65)	2 (1.31)	0.6638
Constipation	0 (0.00)	0 (0.00)	1 (0.65)	0.6645
Acid reflux	0 (0.00)	2 (1.30)	3 (1.96)	0.3372
Anorexia	0 (0.00)	5 (3.25)	4 (2.61)	0.0852
Epigastric distension	1 (0.66)	0 (0.00)	0 (0.00)	0.3312
Epigastric discomfort	2 (1.32)	2 (1.30)	1 (0.65)	0.8746
Infection	3 (1.97)	7 (4.55)	6 (3.92)	0.4419
Cold	2 (1.32)	6 (3.90)	2 (1.31)	0.3408
Pulmonary infection	0 (0.00)	0 (0.00)	1 (0.65)	0.6645
Urinary infection	1 (0.66)	3 (1.95)	3 (1.96)	0.7081
Other	7 (4.61)	20 (12.99)	16 (10.46)	0.0360*
Headache	0 (0.00)	3 (1.95)	2 (1.31)	0.3790
Fatigue	0 (0.00)	1 (0.65)	1 (0.65)	1.0000
Arrhythmia	1 (0.66)	0 (0.00)	3 (1.96)	0.1813
Insomnia	0 (0.00)	0 (0.00)	2 (1.31)	0.2198
Hair loss	1 (0.66)	2 (1.30)	3 (1.96)	0.7910
Mouth ulcer	0 (0.00)	1 (0.65)	1 (0.65)	1.0000
Cough	0 (0.00)	1 (0.65)	1 (0.65)	1.0000
Dry mouth	0 (0.00)	0 (0.00)	1 (0.65)	0.6645
Dry eye	0 (0.00)	1 (0.65)	0 (0.00)	1.0000
Rash	1 (0.66)	4 (2.60)	2 (1.31)	0.5149
Pigmentation	0	0	0	
Leukopenia	2 (1.32)	3 (1.95)	1 (0.65)	0.7910
Abnormal liver function	2 (1.32)	2 (1.30)	0 (0.00)	0.4776
Menstrual disorder	0 (0.00)	1 (0.65)	2 (1.31)	0.6638
Operation	0 (0.00)	0 (0.00)	1 (0.65)	0.6645
Frequent urination	0 (0.00)	2 (1.30)	0 (0.00)	0.3319
Joint swelling and pain	1 (0.66)	0 (0.00)	0 (0.00)	0.3312
Hyperthyroidism	0 (0.00)	1 (0.65)	0 (0.00)	1.0000
Pleural effusion	0 (0.00)	1 (0.65)	0 (0.00)	1.0000
Anemia	0 (0.00)	0 (0.00)	1 (0.65)	0.6645

Data are presented as n (%).

*p < 0.05.

AE, adverse event; IM, integrative medicine; SAE, serious adverse event; TCM, traditional Chinese medicine; WM, Western medicine.

**TABLE 11:** Laboratory parameters (analysis based on safety set).

Adverse event	TCM group (*n* = 152)	WM group (*n* = 154)	IM group (*n* = 153)	*P* Value
Hematologic abnormalities	24 (15.79)	19 (12.34)	13 (8.50)	0.2178
Abnormal WBC count	9 (5.92)	9 (5.84)	7 (4.58)	0.9205
Abnormal HGB level	8 (5.26)	4 (2.60)	4 (2.61)	0.4183
Abnormal platelet count	12 (7.89)	7 (4.55)	4 (2.61)	0.1375
Routine urinalysis	22 (14.47)	34 (22.08)	37 (24.18)	0.2914
Hematuria	13 (8.55)	11 (7.14)	12 (7.84)	0.9922
Proteinuria	6 (3.95)	13 (8.44)	17 (11.11)	0.1982
Bacteriuria	8 (5.26)	20 (12.99)	19 (12.42)	0.0297*
Liver function	11 (7.24)	14 (9.09)	9 (5.88)	0.2996
Abnormal ALT (>40 U/l)	7 (4.61)	10 (6.49)	4 (2.61)	0.2531
Abnormal AST (>35 U/l)	8 (5.26)	8 (5.19)	8 (5.23)	0.4544
Renal function	21 (13.82)	13 (8.44)	9 (5.88)	0.0624
Abnormal creatinine (>84 μmol/l)	14 (9.21)	6 (3.90)	8 (5.23)	0.2291
Abnormal urea nitrogen (>8.2 mmol/l)	7 (4.61)	8 (5.19)	2 (1.31)	0.3191
Abnormal ECG	10 (6.58)	9 (5.84)	10 (6.54)	0.9516

Data are presented as n (%).

*p<0.05.

AE, adverse event; ALT, alanine aminotransferase; AST, aspartate aminotransferase; ECG, electrocardiogram; HGB, hemoglobin; IM, integrative medicine; SAE, serious adverse event; TCM, traditional Chinese medicine; WBC, white blood cell; WM, Western medicine.

## Discussion

The pathogenesis of RA is unknown. The aims of currently recommended treatments are mainly to reduce inflammation, suppress disease activity and delay progression, and prevent bone deformity. RA is classified as “Bi syndrome” in TCM, which refers to a group of diseases involving joint and muscle pain such as RA, osteoarthritis, soft tissue damage, etc. The philosophy of TCM is to maintain overall health and intervene at early stages of disease to prevent progression. However, there is no professional term of “active RA” in TCM. Joint swelling and pain caused by inflammation in RA is considered to be related to dampness, heat, and stasis, which are similar to the active (inflammatory) period of RA (Liu and Jiang, 2020). Therefore, we take the internationally recognized disease activity evaluation standard DAS-28 > 3.2 as the objective identification of RA “damp-heat-stasis syndrome”.

The results of this study demonstrate that TCM treatment is both effective and safe for the treatment of active RA compared to csDMARDs. DAS-28 at 24 weeks was lower in patients treated with QRHXD alone than in those treated with MTX plus HCQ; thus, Qingre Huoxue treatment did not have the expected therapeutic advantage over standard RA treatments. However, it is worth noting that the score was lower in the IM group than in the WM group at the end of the treatment period, suggesting that QRHXD can enhance the efficacy of csDMARDs. It was previously reported that QRHXD is suitable for the treatment of “damp-heat-stasis” as it delayed disease activity by reducing inflammation and potentially conferred bone protection (Jiang et al., 2012). In line with the comprehensive treatment approaches used in TCM, patients received QRHXD and QRHXEP during the active period of RA. QRHXEP should not be ignored in efficacy assessments; however, as a topical formulation, its effects begin to decline after about 4 weeks and last only 12 weeks; thus, by the end of the 24-week study period, QRHXD was likely responsible for the observed therapeutic effects of TCM.

In order to standardize the evaluation of “damp-heat-stasis syndrome”, we used DAS-28 > 3.2 to define the active period of RA. DAS-28 (Prevoo et al., 1995), which is based on a count of 28 swollen and tender joints and ranges from 0 to 9.4, can be used to objectively evaluate a patient’s response to treatment (Fransen and van Riel, 2009). The European League Against Rheumatism response criteria combine the DAS-28 score at the time of evaluation with the change in DAS-28 score between two time points, which is a more useful measure of treatment response (van Gestel et al., 1998). Combining DAS-28 score and ACR-20/50/70 can provide more information on the therapeutic benefit of TCM for the treatment of RA beyond its effect on joints (He et al., 2007; He et al., 2008; He et al., 2014).

This is the first registered randomized controlled trial investigating the efficacy and safety of TCM Qingre Huoxue treatment compared to csDMARDs in active RA. Given the large sample size and the involvement of numerous medical centers, the results are representative of the Chinese population. We found that the TCM Qingre Huoxue treatment improved DAS-28 from baseline to week 24, whether it was administered alone or in combination with MTX and HCQ. In the FAS, ACR-20 was 73.04%, ACR-50 was 40.87%, and ACR-70 was 20.87% with TCM Qingre Huoxue treatment at 24 weeks; the TCM efficacy at the end of the study was only slightly lower than that observed in the WM and IM groups. In this study, we found that Qingre Huoxue treatment can improve DAS-28 score and ACR-20/50/70 by improving joint tenderness, joint swelling, ESR and CRP. According to other studies (He et al., 2007; He et al., 2014), ACR-20 and ACR-50 responses with WM treatment were higher at 24 weeks than in the TCM group. Whether a therapeutic advantage of TCM over MTX and HCQ will be revealed with a longer follow-up period remains to be determined. At 24 weeks, ACR-20/50/70 was similar in patients treated with QRHXD alone to in those treated with MTX plus HCQ. In clinical practice, we often use QRHXD as a prescription for RA patients from active stage to remission stage, whether a longer follow-up might show further benefit for QRHXD treatment can only be answered with such a longer study.

In our study, subjects in the WM group received a combination the csDMARDs MTX and HCQ (Singh et al., 2016). In China, most rheumatologists choose to use two kinds of csDMARDs combined with drugs in the treatment of active RA patients, and MTX alone is only used for RA patients with mild condition. The combination of MTX and HCQ is the most commonly used prescription. Therefore, MTX + HCQ is more representative in China. With this treatment, DAS-28 score in the FAS decreased and ACR-20/50/70 responses were 80.17, 47.93, and 22.31%, respectively, at 24 weeks, which is in line with results obtained in other studies (O’Dell et al., 2002; Hua et al., 2020; Zhang et al., 2020). In one study, an ACR-20 of 71.4% was reported after 24 weeks with MTX treatment alone, which was lower than the response achieved with the combination of MTX and HCQ (Westhovens et al., 2021). In our study, there were no differences in ACR-20/50/70 among the three groups at 24 weeks.

The TCM group had the lowest rate of TRAEs. Patients in the WM group were more likely to discontinue treatment due to TRAEs, and reported significantly higher rates of gastrointestinal discomfort (e.g., nausea) and bacteriuria along with the IM group compared to patients treated with TCM. These data suggest that TCM is more suitable for RA patients with poor gastrointestinal function or urinary tract bacterial infection, especially as the use of TCM did not increase the risk of infections (Table 10).

## Conclusion

The results of this study provide evidence that Qingre Huoxue treatment (QRHXD plus QRHXEP) is effective for the treatment of patients with active RA, with a better safety profile than MTX and HCQ. While the efficacy of QRHXD alone was lower than that of the two csDMARDs, combining QRHXD with MTX and HCQ yielded the greatest improvement in disease activity. The efficacy of QRHXD alone was similar to that of MTX + HCQ in achieving ACR-20/50/70. Thus, QRHXD is a useful adjunct that should be considered as a viable option in the management of RA.

## Data Availability

The original contributions presented in the study are included in the article/Supplementary Material, further inquiries can be directed to the corresponding authors.

## References

[B1] Abbasi M. Mousavi M. J. Jamalzehi S. Alimohammadi R. Bezvan M. H. Mohammadi H. (2019). Strategies toward Rheumatoid Arthritis Therapy; the Old and the New. J. Cel. Physiol. 234, 10018–10031. 10.1002/jcp.27860 30536757

[B2] Aletaha D. Neogi T. Silman A. J. Funovits J. Felson D. T. Bingham C. O. 3rd (2010). 2010 Rheumatoid Arthritis Classification Criteria: An American College of Rheumatology/European League against Rheumatism Collaborative Initiative. Arthritis Rheum. 62, 2569–2581. 10.1002/art.27584 20872595

[B3] Arnett F. C. Edworthy S. M. Bloch D. A. McShane D. J. Fries J. F. Cooper N. S. (1988). The American Rheumatism Association 1987 Revised Criteria for the Classification of Rheumatoid Arthritis. Arthritis Rheum. 31, 315–324. 10.1002/art.1780310302 3358796

[B4] Bruce B. Fries J. F. (2005). The Health Assessment Questionnaire (HAQ). Clin. Exp. Rheumatol. 23, S14–S18. 16273780

[B5] Chinese Pharmacopoeia Commission (2005). [Pharmacopoeia of the People’s Republic of China]. Beijing: People’s Medical Publishing House.

[B6] Chinese Rheumatology Association (2018). 2018 Chinese Guideline for the Diagnosis and Treatment of Rheumatoid Arthritis. Zhonghua Nei Ke Za Zhi 57, 242–251. article in Chinese. 10.3760/cma.j.issn.0578-1426.2018.04.004 29614581

[B7] China Association of Chinese Medicine. (2017). [Guidelines for Diagnosis and Treatment of Rheumatoid Arthritis Based on Combination of Disease and Syndrome]. Chin. Med. (Cptcm) 10, 1–9. article in Chinese.

[B8] Felson D. T. Anderson J. J. Boers M. Bombardier C. Furst D. Goldsmith C. (1995). American College of Rheumatology Preliminary Definition of Improvement in Rheumatoid Arthritis. Arthritis Rheum. 38, 727–735. 10.1002/art.1780380602 7779114

[B9] Fransen J. van Riel P. L. C. M. (2009). The Disease Activity Score and the EULAR Response Criteria. Rheum. Dis. Clin. North America 35, 745–757. 10.1016/j.rdc.2009.10.001 19962619

[B10] Giraud E. L. Jessurun N. T. van Hunsel F. P. A. M. van Puijenbroek E. P. van Tubergen A. Ten Klooster P. M. (2020). Frequency of Real-World Reported Adverse Drug Reactions in Rheumatoid Arthritis Patients. Expert Opin. Drug Saf. 19, 1617–1624. 10.1080/14740338.2020.1830058 32990050

[B11] He Y.-t. Ou A.-h. Yang X.-b. Chen W. Fu L.-y. Lu A.-p. (2014). Traditional Chinese Medicine versus Western Medicine as Used in China in the Management of Rheumatoid Arthritis: A Randomized, Single-Blind, 24-week Study. Rheumatol. Int. 34, 1647–1655. 10.1007/s00296-014-3010-6 24760484

[B12] He Y. Lu A. Zha Y. Tsang I. (2008). Differential Effect on Symptoms Treated with Traditional Chinese Medicine and Western Combination Therapy in RA Patients. Complement. Therapies Med. 16, 206–211. 10.1016/j.ctim.2007.08.005 18638711

[B13] He Y. Lu A. Zha Y. Yan X. Song Y. Zeng S. (2007). Correlations between Symptoms as Assessed in Traditional Chinese Medicine (TCM) and ACR20 Efficacy Response. J. Clin. Rheumatol. 13, 317–321. 10.1097/RHU.0b013e31815d019b 18176139

[B14] Hua L. Du H. Ying M. Wu H. Fan J. Shi X. (2020). Efficacy and Safety of Low-Dose Glucocorticoids Combined with Methotrexate and Hydroxychloroquine in the Treatment of Early Rheumatoid Arthritis. Medicine (Baltimore) 99, e20824. 10.1097/MD.0000000000020824 32629668 PMC7337402

[B15] Jiang Q. Zhou X.-y. Wang L. Yu W. Wang P. Cao W. (2012). A One-Year Evaluation of Radiographic Progression in Patients with Rheumatoid Arthritis Treated by Qingre Huoxue Decoction. Chin. J. Integr. Med. 18, 256–261. 10.1007/s11655-011-0793-0 21853348

[B16] Jiao J. Tang X. Gong X. Yin H. Jiang Q. Wei C. (2019). Effect of Cream, Prepared with *Tripterygium Wilfordii* Hook F and Other Four Medicinals, on Joint Pain and Swelling in Patients with Rheumatoid Arthritis: A Double-Blinded, Randomized, Placebo Controlled Clinical Trial. J. Tradit. Chin. Med. 39, 89–96. 32186028

[B17] Jin S. Li M. Li M. Fang Y. Li Q. Liu J. (2017). Chinese Registry of Rheumatoid Arthritis (CREDIT): II. Prevalence and Risk Factors of Major Comorbidities in Chinese Patients with Rheumatoid Arthritis. Arthritis Res. Ther. 19, 251. 10.1186/s13075-017-1457-z 29141688 PMC5688621

[B18] Lau C. S. Chia F. Dans L. Harrison A. Hsieh T. Y. Jain R. (2019). 2018 Update of the APLAR Recommendations for Treatment of Rheumatoid Arthritis. Int. J. Rheum. Dis. 22, 357–375. 10.1111/1756-185X.13513 30809944

[B19] Liu W. X. Jiang Q. (2020). Exploration of the Pathogenesis Theory of “Dampness-heat-stasis” in Rheumatoid Arthritis. J. Tradit. Chin. Med. 61, 2148–2153. article in Chinese.

[B20] Lu M.-C. Livneh H. Chiu L.-M. Lai N.-S. Yeh C.-C. Tsai T.-Y. (2019). A Survey of Traditional Chinese Medicine Use Among Rheumatoid Arthritis Patients: A Claims Data-Based Cohort Study. Clin. Rheumatol. 38, 1393–1400. 10.1007/s10067-018-04425-w 30671749

[B21] Lu Z. Z. Jiao S. D. (1996). Diagnostic Criteria of National Integrated Traditional and Western Academic Conference on Rheumatism (1988). Beijing, China: People Public Health Publishing Company, Vol. 456, 16..

[B22] O'Dell J. R. Leff R. Paulsen G. Haire C. Mallek J. Eckhoff P. J. (2002). Treatment of Rheumatoid Arthritis with Methotrexate and Hydroxychloroquine, Methotrexate and Sulfasalazine, or a Combination of the Three Medications: Results of a Two-Year, Randomized, Double-Blind, Placebo-Controlled Trial. Arthritis Rheum. 46, 1164–1170. 10.1002/art.10228 12115219

[B23] Pincus T. Yazici Y. Sokka T. Aletaha D. Smolen J. S. (2003). Methotrexate as the “Anchor Drug” for the Treatment of Early Rheumatoid Arthritis. Clin. Exp. Rheumatol. 21, S179–S185. 14969073

[B24] Prevoo M. L. L. Van'T Hof M. A. Kuper H. H. van Leeuwen M. A. van de Putte L. B. A. van Riel P. L. C. M. (1995). Modified Disease Activity Scores that Include Twenty-Eight-Joint Counts Development and Validation in a Prospective Longitudinal Study of Patients with Rheumatoid Arthritis. Arthritis Rheum. 38, 44–48. 10.1002/art.1780380107 7818570

[B25] Singh J. A. Saag K. G. Bridges S. L. Jr Akl E. A. Bannuru R. R. Sullivan M. C. (2016). 2015 American College of Rheumatology Guideline for the Treatment of Rheumatoid Arthritis. Arthritis Rheumatol. 68, 1–26. 10.1002/art.39480 26545940

[B26] Smolen J. S. Landewé R. B. M. Bijlsma J. W. J. Burmester G. R. Dougados M. Kerschbaumer A. (2020). EULAR Recommendations for the Management of Rheumatoid Arthritis with Synthetic and Biological Disease-Modifying Antirheumatic Drugs: 2019 Update. Ann. Rheum. Dis. 79, 685–699. 10.1136/annrheumdis-2019-216655 31969328

[B27] van der Woude D. van der Helm-van Mil A. H. M. (2018). Update on the Epidemiology, Risk Factors, and Disease Outcomes of Rheumatoid Arthritis. Best Pract. Res. Clin. Rheumatol. 32, 174–187. 10.1016/j.berh.2018.10.005 30527425

[B28] van Gestel A. M. Haagsma C. J. van Riel P. L. C. M. (1998). Validation of Rheumatoid Arthritis Improvement Criteria that Include Simplified Joint Counts. Arthritis Rheum. 41, 1845–1850. 10.1002/1529-0131(199810)41:10<1845::aid-art17>3.0.co;2-k 9778226

[B29] Wells G. Becker J.-C. Teng J. Dougados M. Schiff M. Smolen J. (2009). Validation of the 28-joint Disease Activity Score (DAS28) and European League against Rheumatism Response Criteria Based on C-Reactive Protein against Disease Progression in Patients with Rheumatoid Arthritis, and Comparison with the DAS28 Based on Erythrocyte Sedimentation Rate. Ann. Rheum. Dis. 68, 954–960. 10.1136/ard.2007.084459 18490431 PMC2674547

[B30] Westhovens R. Rigby W. F. C. van der Heijde D. Ching D. W. T. Stohl W. Kay J. (2021). Filgotinib in Combination with Methotrexate or as Monotherapy versus Methotrexate Monotherapy in Patients with Active Rheumatoid Arthritis and Limited or No Prior Exposure to Methotrexate: The Phase 3, Randomised Controlled FINCH 3 Trial. Ann. Rheum. Dis., 10.1136/annrheumdis-2020-219213 PMC8142453 33452004

[B31] Xing Q. Fu L. Yu Z. Zhou X. (2020). Efficacy and Safety of Integrated Traditional Chinese Medicine and Western Medicine on the Treatment of Rheumatoid Arthritis: A Meta-Analysis. Evidence-Based Complement. Altern. Med. 2020, 1–15. 10.1155/2020/4348709 PMC7154968 32328130

[B32] Zhang L. Chen F. Geng S. Wang X. Gu L. Lang Y. (2020). Methotrexate (MTX) Plus Hydroxychloroquine versus MTX Plus Leflunomide in Patients with MTX-Resistant Active Rheumatoid Arthritis: A 2-year Cohort Study in Real World. J. Inflamm. Res. Vol. 13, 1141–1150. 10.2147/JIR.S282249 33376379 PMC7755368

[B33] Zhang S. Wang X. Li C. An Y. Zhou Y. S. Liu J. (2013). Investigation of Hydroxychloroquine Use in Rheumatoid Arthritis Patients in China. Chin. J. Rheumatol. 17, 585–590. article in Chinese.

[B34] Zheng X. Y. (2002). Clinical Research Criteria on Treating RA by Chinese Herbs. Med. Sci. Technol. Press. China 5, 115–119.

