# Supplementary material for: Traditional Chinese Medicine Qingre Huoxue Treatment vs. the Combination of Methotrexate and Hydroxychloroquine for Active Rheumatoid Arthritis: A Multicenter, Double-Blind, Randomized Controlled Trial
Source: Front Pharmacol. 2021 May 25;12:679588. doi: 10.3389/fphar.2021.679588 (PMC8186316; doi:10.3389/fphar.2021.679588)
Supplement: Supplementary file 1 [file DataSheet1.docx]

Supplementary Material

**Phytochemical Characterization of Qingre Huoxue decoction (QRHXD), Qingre Huoxue external preparation (QRHXEP), QRHXD placebo, QRHXEP placebo**

# 1. Method: LC/MS/MS method

# 2.Sample preparation

TCM Qingre Huoxue decoction (granules) 1g, add 50ml water, ultrasonic for 30min, filter with 0.2 μm filter membrane (Acrodisc 13mm Minispike with 0.2μm GHP Membrane 1000/pk), filter into Shimadzu injection vial.

TCM Qingre Huoxue external preparation (gel) 0.5g, water 50ml, vortex, filter with 0.2 μm filter membrane (Acrodisc 13mm Minispike with 0.2μm GHP Membrane 1000/pk), filter into Shimadzu injection vial.

TCM Qingre Huoxue decoction placebo (granules) 1g, add 50ml water, ultrasonic for 30min, filter with 0.2 μm filter membrane (Acrodisc 13mm Minispike with 0.2μm GHP Membrane 1000/pk), filter into Shimadzu injection vial.

TCM Qingre Huoxue external preparation placebo (gel) 0.5g, water 50ml, vortex, filter with 0.2 μm filter membrane (Acrodisc 13mm Minispike with 0.2μm GHP Membrane 1000/pk), filter into Shimadzu injection vial.

# 3.LC conditions

system: SHIMADZU UPLC (Valve unit, Degassing unit, Liouqid Chromatograph , Autosampler , Communications bus module , Prominence UV/VIS Detector,Prominence Column oven ),

Column: ACQUITY UPLC® HSS T3（φ2.1×100mm，1.8μm）

Column temperature: 30 °C

Injection volume: 2 µL

Flow rate: 0.3 mL/min

Mobile phase A:Acetonitrile

Mobile phase B: Water + 0.1% acetic acid

**4.MS conditions**

MS system: Triple TOF 5600^+^ (AB SCIEX, USA),

Ionization: APCI positive,

Ion Source Gas1:50,

Ion Source Gas2: 50,

Curtain Gas:35,

Temperature: 500.0,

Declustering Potential:80

Collision Energy:45±25


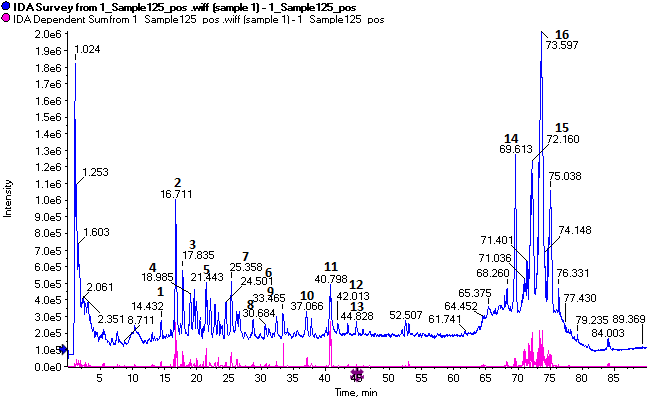


**Figure 1.**LC/MS/MS Chromatograms of representative compounds in QRHXD


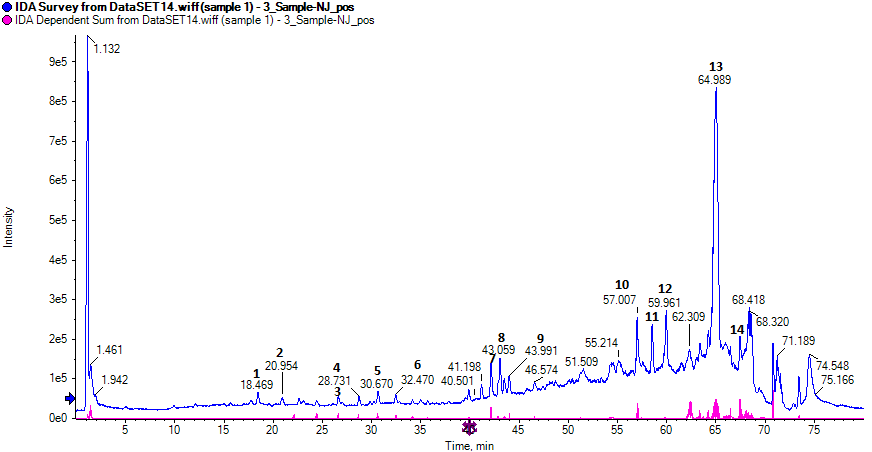


**Figure 2.** LC/MS/MS Chromatograms of representative compounds in QRHXEP


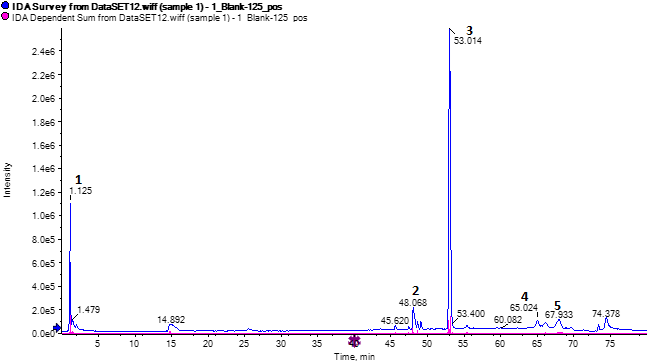


**Figure 3.** LC/MS/MS Chromatograms of representative compounds in QRHXD placebo


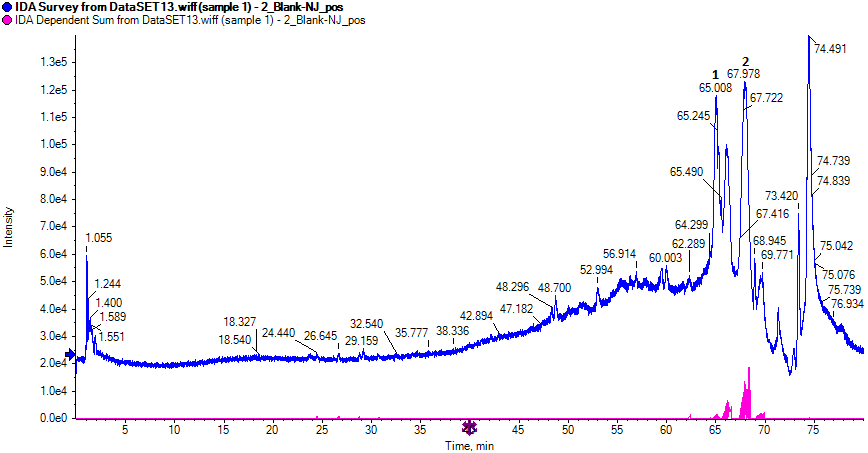


**Figure 4.** LC/MS/MS Chromatograms of representative compounds in QRHXEP placebo

**Table 1.** The gradient

| Time | Mobile phase A | Mobile phase B |
| --- | --- | --- |
| 0 | 1 | 99 |
| 5 | 1 | 99 |
| 15 | 10 | 90 |
| 35 | 20 | 80 |
| 60 | 40 | 60 |
| 75 | 100 | 0 |
| 90 | 100 | 0 |

**Table 2.**Chemical profiling of QRHXD

| No. | Retention time (min) | Compound |
| --- | --- | --- |
| 1 | 14.432 | Neochlorogenic acid |
| 2 | 16.711 | Sinomenine |
| 3 | 17.835 | Chlorogenic acid |
| 4 | 18.985 | Cryptochlorogenic acid |
| 5 | 21.443 | Phellodendrine |
| 6 | 24.501 | Dictamnoside L |
| 7 | 25.358 | 6,7-Dimethoxycoumarin |
| 8 | 30.684 | Sarmentoloside |
| 9 | 33.465 | Aempferol-3-O-rutinoside |
| 10 | 37.066 | Vitexin-2-O-rhamnoside |
| 11 | 40.798 | Berberine hydrochloride |
| 12 | 42.013 | Blinin |
| 13 | 44.828 | 7β-Hydroxy-3,11,15,23-tetraoxolanosta-8,20E(22)-dien-26-methyl gallate |
| 14 | 69.613 | Diosgenin Glucoside |
| 15 | 72.160 | Neoandrographolide |
| 16 | 73.597 | C19-Obtusilactone dimer |

**Table 3.** Chemical profiling of QRHXEP

| No. | Retention time (min) | Compound |
| --- | --- | --- |
| 1 | 18.469 | m-Hydroxybenzaldehyde |
| 2 | 20.954 | 3-Hydroxymorindone |
| 3 | 26.652 | ( E ) -3,7-dimethyl-1-O- [ α-L-rhamnopyranosyl- ( 1 → 6 ) -β-D-glucopyranosyl-octacarbon-2-en-7-ol |
| 4 | 28.731 | 2α-acetoxy-4β-hydroxy-6α-dangguioyloxy-10β-cinnamoyloxy-carrot-8-ene |
| 5 | 30.670 | Adouetine Y |
| 6 | 32.470 | Emodin anthrone |
| 7 | 42.157 | 1,4-Naphthoquinone |
| 8 | 43.059 | 5-Methoxy-8-hydroxy-psoralen |
| 9 | 43.991 | Anthraquinone |
| 10 | 57.007 | Coumarin |
| 11 | 58.515 | 5-(3-Buten-1-ynyl)-2,2'-bithienyl |
| 12 | 59.961 | γ-Dodecalactone |
| 13 | 64.989 | Triptocalline A |
| 14 | 67.457 | Triptotriterpenic acid C |

**Table 4.** Chemical profiling of QRHXD placebo

| No. | Retention time (min) | Compound |
| --- | --- | --- |
| 1 | 1.125 | 3,6-Anhydrogalactose |
| 2 | 48.068 | Isolimocitrol-3-β-D-glucoside |
| 3 | 53.014 | Tamarix ( lutein ) -3 - O-β-D-glucopyranoside 7 - O-β-D-glucopyranoside |
| 4 | 65.024 | Tetradecenoic acid B |
| 5 | 67.933 | 3β,19α-Dihydroxyurs-12-en-28-oic acid 28-β-D-glucopyranosyl es |

**Table 5.** Chemical profiling of QRHXEP placebo

| No. | Retention time (min) | Compound |
| --- | --- | --- |
| 1 | 65.008 | Tetradecenoic acid B |
| 2 | 67.978 | erythro-2-Hydroxy-2-(1-hydroxyethyl)-4-methylpentanoic acid |
